# CD11b^−^CD27^−^ NK Cells Are Associated with the Progression of Lung Carcinoma

**DOI:** 10.1371/journal.pone.0061024

**Published:** 2013-04-02

**Authors:** Jing Jin, Binqing Fu, Xinyu Mei, Ting Yue, Rui Sun, Zhigang Tian, Haiming Wei

**Affiliations:** 1 Institute of Immunology, School of Life Sciences, University of Science and Technology of China, Hefei, Anhui, China; 2 Hefei National Laboratory for Physical Sciences at Microscale, University of Science and Technology of China, Hefei, Anhui, China; 3 Anhui Provincial Hospital, Hefei, Anhui, China; University of Manitoba, Canada

## Abstract

NK cells are a major component of the antitumour immune response that limits tumour progression. However, it has been reported that tumour-infiltrating NK (TINK) cells from patients with non-small-cell lung carcinoma (NSCLC) exhibit profound defects in degranulation and IFN-γ production. In support of this notion, we report a novel mechanism associated with tumour escape from NK cell-mediated antitumour immunity in lung carcinoma. In this study, we investigated the phenotypic profile of TINK cells based on the expression of the NK-cell maturation markers CD11b and CD27. Interestingly, we found a substantial CD11b^−^CD27^−^ (DN) NK-cell population harboured within the tumour tissues. The presence of this CD11b^−^CD27^−^ NK subset indicated that the TINK cells were of an immature and inactive phenotype. Remarkably, we determined that the presence of DN NK cells had an impact on the clinical outcomes of patients with NSCLC, as the frequency of tumour-infiltrating DN NK cells was positively correlated with the tumour stage and tumour size. We further used a murine Lewis lung cancer (LLC) model to confirm the correlation between the frequency of tumour-infiltrating DN NK cells and the progression of lung carcinoma. Together, our findings demonstrate that the tumour microenvironment may render TINK cells less tumouricidal and thereby contribute to cancer progression.

## Introduction

Lung cancer is the leading cause of cancer-related deaths worldwide [1]. Innovative and efficacious therapeutic strategies, including immunotherapies, are therefore urgently needed. A thorough understanding of anti-tumour immune responses and especially of the role of NK cells in this process is necessary [2]–[6]. NK cells are innate lymphocytes that have a natural ability to recognise and kill aberrant cells, including cancer cells [7]–[10]. However, elegant studies have provided evidence that NSCLC-infiltrating NK cells exhibit profound defects in degranulation and IFN-γ production [11]. These defects are associated with coordinated changes in the NK-cell receptor repertoire, suggesting a local tumour-induced impairment of NK-cell function. These findings suggest that it is not the quantity but the quality of intratumoural NK cells that accounts for their dysfunction in NSCLC. Notably, that study also revealed that intratumoural NK cells expressed dramatically lower levels of killer-cell immunoglobulin-like receptor (KIR) than did peripheral blood NK (pNK) cells from the same patients. Similarly, another study demonstrated the loss of KIR expression on intratumoural NK cells when compared with NK cells from the peritumoural lung tissue [12]. Interestingly, the lack of KIR expression on tumour-infiltrating NK (TINK) cells is also observed in human breast tumours [13], whereas KIR is present on NK cells infiltrating healthy mammary tissue (Mt-NK cells). This finding suggests that TINK cells, like non-educated cells, have no cytotoxic capacity [14], [15]. This study also indicated that the phenotype of TINK cells was characteristic of immature and nonfunctional NK cells [13]. In addition, the terminal differentiation of NK cells is characterised by the appearance of KIRs and decreased NKG2A expression [16], [17]. Accordingly, the study describing the loss of KIR expression on TINK cells from patients with breast cancer suggests that a strong inhibitory environment, such as the tumour microenvironment, can reorient or reverse the transcriptional program of NK cell maturation toward a nonreactive self-tolerant profile [13]. In support of this hypothesis, several recent studies have shown that the NK-cell developmental program is not entirely fixed and that mature NK cells can be re-educated by their environment [18]–[20]. Hence, it is likely that the tumour microenvironment may have a negative impact on NK-cell maturation.

NK cells are considered a heterogeneous population and may be divided into different subsets [21], [22]. Human NK cells can be subdivided into 4 differentiation stages based on surface expression of CD34, CD117 and CD94 [23], [24]. Human NK cells are traditionally defined as CD56^+^CD3^−^ cells. The majority of these cells belong to the most mature population (stage 4; CD34^−^CD117^−^CD94^+/−^), and CD56 is only highly expressed during stage 4. The heterogeneity of stage 4 NK cells has garnered additional investigation. Based on the expression levels of CD56, human NK cells can be subdivided into two main subsets: CD56^bright^ NK cells have predominantly immunoregulatory functions mediated by their potent cytokine production capacity, whereas CD56^dim^ NK cells have a marked cytotoxic function [25]–[28]. Despite the knowledge of these maturation markers, the functional differences between these populations remain poorly understood. Thus, much effort has recently been devoted toward subdividing human NK cells into functionally distinct subpopulations. Recent studies have shown that CD27, a member of the tumour necrosis factor receptor superfamily, is an important marker that can be used to define NK-cell subsets [29], [30]. The surface density of CD27 and CD11b can be used to divide murine NK cells into 4 subsets that define different levels of maturation [31], [32]. Based on the findings in previous studies, we hypothesised that the expression of CD11b and CD27 may similarly define distinct populations of human NK cells [33]. Despite advances in understanding NK-cell development, the specific NK-cell subsets harboured within tumours and the mechanisms that might account for the NK-cell infiltration of tumours have not yet been defined.

A recent study revealed that mature CD27^high^ NK cells are the predominant NK-cell subpopulation that accumulates within the tumour microenvironment. However, this work was performed in an early-developing murine MCA205 fibrosarcoma tumour model [34]. The impact of mouse studies on the clinical monitoring of human NK-cell function and on the design of improved therapeutics for cancer has been limited. Hence, we aimed to identify whether the same NK-cell subset was present in the human tumour microenvironment and to precisely characterise NK-cell differentiation within human tumours. To this end, we investigated the expression of CD11b and CD27 on NK cells isolated from primary human NSCLC tumour specimens and peripheral blood from the same patients as well as peripheral blood from healthy control subjects. In agreement with the findings of previous studies, nearly all of the pNK cells from NSCLC patients and healthy control subjects were CD11b^+^CD27^−^ (CD11b^+^SP) NK cells. However, TINK cells isolated from patients with NSCLC contained CD11b^−^CD27^−^ (DN) NK cells in addition to CD11b^+^SP NK cells. Furthermore, we demonstrated that the presence of the CD11b^−^CD27^−^ population was associated with clinical outcomes in patients with NSCLC, as the frequency of CD11b^−^CD27^−^ cells within the tumour positively correlated with the tumour stage and tumour size. We further verified the correlation between the frequency of CD11b^−^CD27^−^ TINK cells and tumour progression in a murine Lewis lung cancer (LLC) model. Taken together, our findings provide direct evidence that CD11b^−^CD27^−^ NK cells, which have been defined as immature NK cells in previous studies, accumulate within tumours, suggesting that the tumour microenvironment may render TINK cells less tumouricidal and thereby contribute to cancer progression. Importantly, our results reveal a novel mechanism underlying NK-cell dysfunction during antitumour immune responses.

## Results

### DN NK cells are present within the TINK-cell population

**Figure 1:**
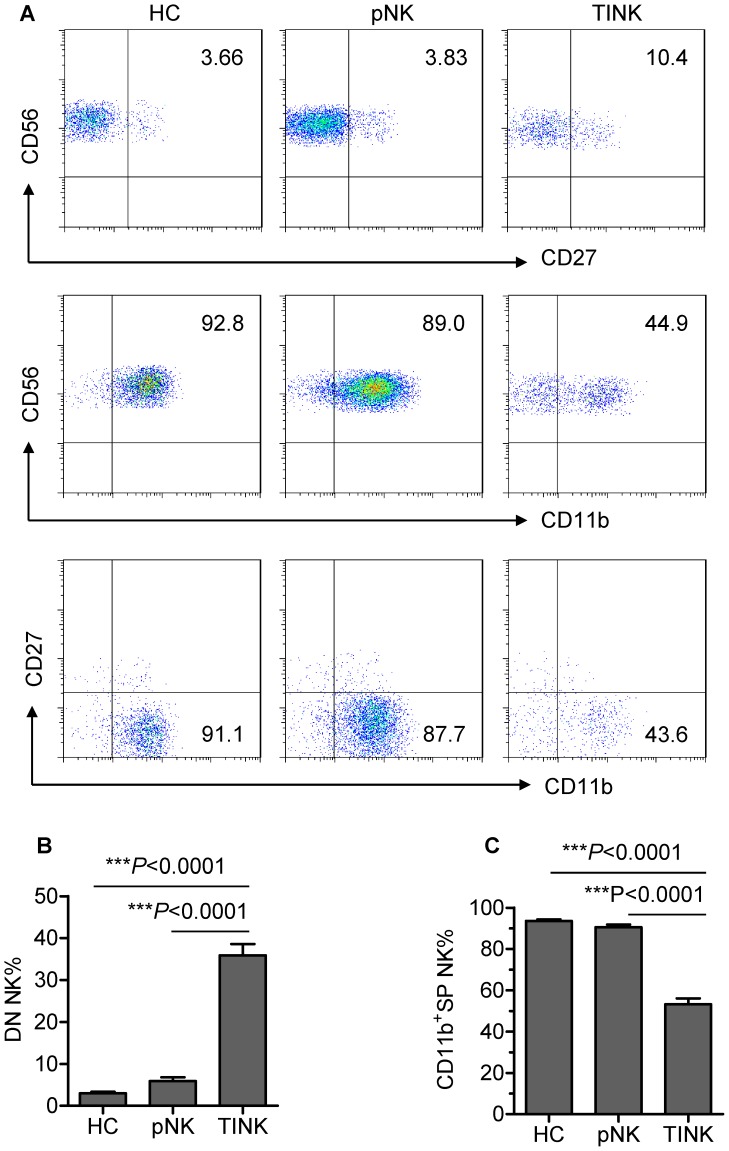
DN NK cells are present within the TINK-cell population. (A) Representative flow cytometry analysis of the expression of CD27 and CD11b on gated CD56^+^CD3^−^ TINK cells as compared with pNK cells from autologous patients and healthy control subjects. Dot plots were gated on live NK cells using a lymphocyte gate based on forward scatter versus side scatter and an NK-cell gate identifying CD56^+^CD3^−^ cells. Quadrants depicted were set on isotype controls. (B) The frequency of DN NK cells within the TINK-cell population as compared with that within the pNK-cell populations from autologous patients and healthy control subjects (n = 35; mean±SEM). (C) The frequency of CD11b^+^SP NK cells within the TINK-cell population as compared with that within the pNK-cell populations from autologous patients and healthy control subjects (n = 35; mean±SEM).

To determine the maturation status of TINK cells, we compared the expression of CD11b and CD27 on TINK cells with that on pNK cells from autologous patients and healthy control subjects. Approximately 10% of TINK cells were CD27^+^, whereas less than 5% of pNK cells from autologous patients and healthy control subjects were CD27^+^. Interestingly, TINK cells exhibited a significant reduction in CD11b expression when compared with the other two types of NK cells (Fig. 1A). Furthermore, we detected a DN (CD11b^−^CD27^−^) NK-cell subset that accounted for approximately 35% of the TINK population (Fig. 1B). By contrast, the proportion of CD11b^+^SP (CD11b^+^CD27^−^) NK cells was lower within the TINK population than within the pNK population (Fig. 1C). These data demonstrate, for the first time, that a large population of DN NK cells exists within the TINK-cell population in human tumours, suggesting an immature phenotype of TINK cells.

### TINK cells display an immature and inactive phenotype

**Figure 2:**
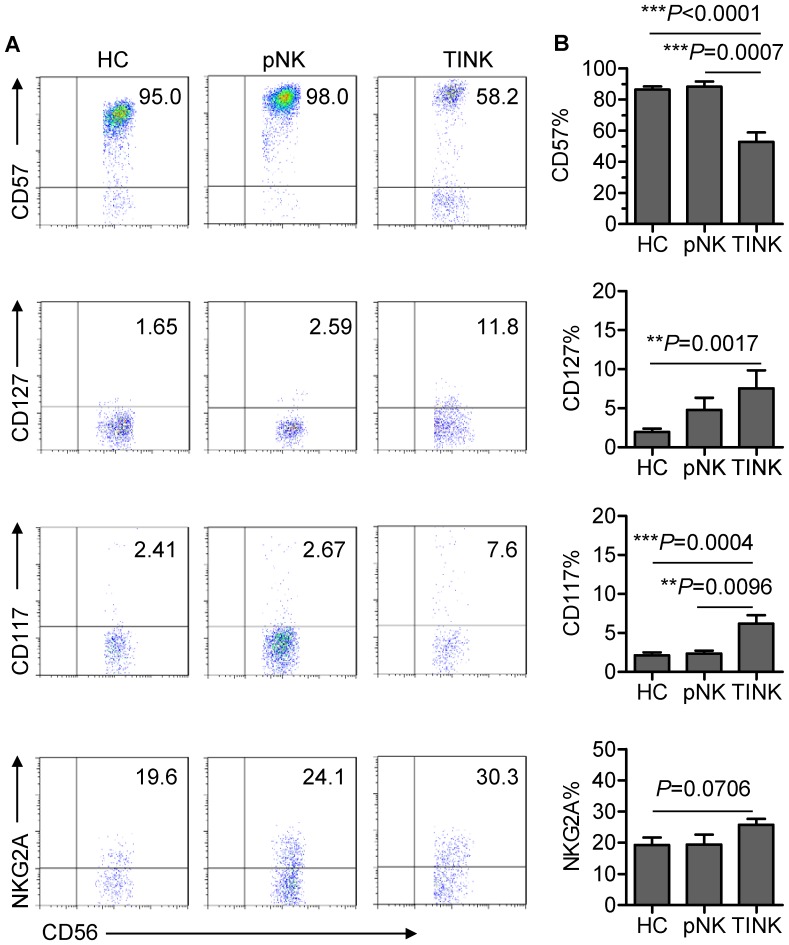
TINK cells display an immature phenotype. (A) Representative flow cytometry analysis of the expression of NK-cell maturation receptors (CD57, CD127, CD117 and NKG2A) on gated CD56^+^CD3^−^ TINK cells as compared with that on pNK cells from autologous patients and healthy control subjects. Quadrants depicted were set on isotype controls. (B) The frequency of CD57^+^, CD127^+^, CD117^+^ and NKG2A^+^ NK cells within the above-mentioned three NK-cell populations (n = 15; mean±SEM).

To determine whether TINK cells have an immature phenotype, we investigated the expression of NK-cell receptors associated with maturity on CD56^+^CD3^−^ TINK cells and pNK cells from autologous patients and healthy control subjects. Downregulation of CD57, which is expressed on highly mature NK cells [35], was observed on TINK cells, whereas no change in CD57 expression was observed on pNK cells from autologous patients and healthy control subjects (Fig. 2A and B). Conversely, CD127 and CD117, receptors that are virtually absent on traditional mature NK cells [21], were clearly expressed on TINK cells (Fig. 2A and B). Moreover, NKG2A, an inhibitory receptor reported to be highly expressed by immature NK cells, was slightly upregulated on TINK cells when compared with the other two types of NK cells (Fig. 2A and B). Collectively, these results demonstrate that TINK cells display an immature phenotype.

**Figure 3:**
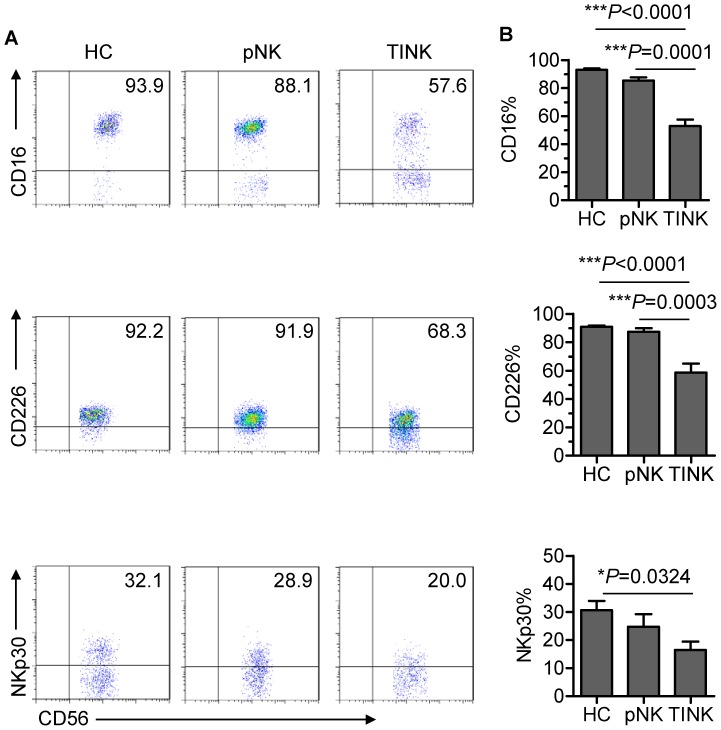
TINK cells display an inactive phenotype. (A) Representative flow cytometry analysis of the expression of NK-cell activation receptors (CD16, CD226 and NKp30) on gated CD56^+^CD3^−^ TINK cells as compared with that on pNK cells from autologous patients and healthy control subjects. Quadrants depicted were set on isotype controls. (B) The frequency of CD16^+^, CD226^+^ and NKp30^+^ NK cells within the above-mentioned three NK-cell populations (n = 15; mean±SEM).

We next examined the expression of NK-cell receptors that have been reported to be involved in NK-cell activation [36], [37]. Previous reports have demonstrated that NK cells infiltrating human non-small-cell lung cancer are enriched in CD16^−^ cells[11], [12]. Similarly, we found that the expression of CD16 (Fcγ receptor III) was dramatically reduced on TINK cells from NSCLC patients as compared with pNK cells from autologous patients and healthy control subjects (Fig. 3A and B). CD16 facilitates antibody-dependent cellular cytotoxicity and is used as an NK-cell maturation marker [38]. Hence, reduced expression of CD16 on TINK cells further confirms that TINK cells are phenotypically immature. In addition, CD226 and NKp30 were expressed at significantly lower levels on TINK cells than on pNK cells (Fig. 3A and B). Together, these data confirm that TINK cells exhibit an inactive phenotype, supporting previous reports of their poor cytotoxic capacity [11], [12]. Based on these results, we hypothesise that TINK-cell dysfunction is associated with their immature phenotype, as the TINK-cell population contains a large subset of DN NK cells.

### DN NK cells account for the immature characteristics of TINK cells

**Figure 4:**
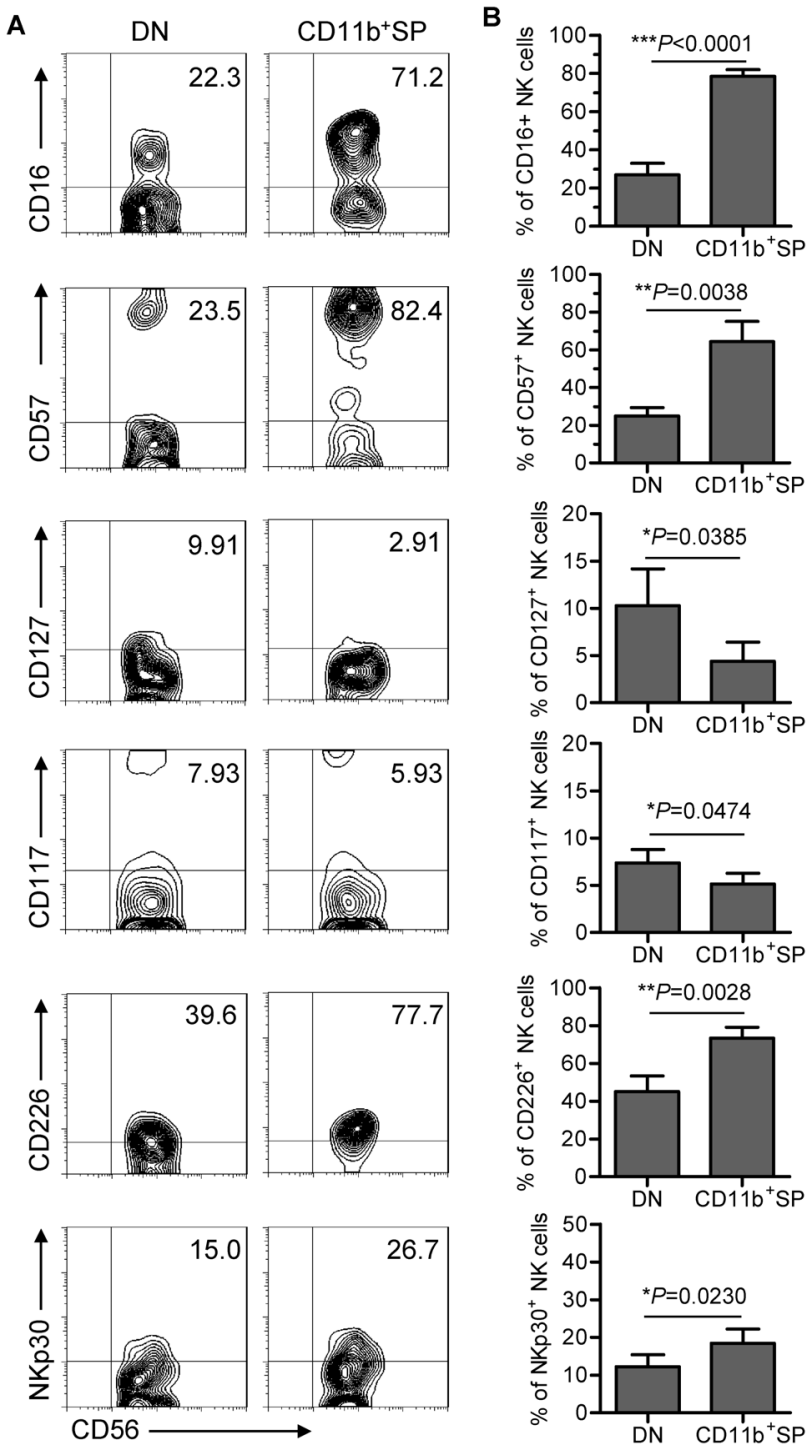
The DN NK subset in TINK cells accounts for the immature phenotype. (A) Representative flow cytometry analysis of the expression of various surface molecules (CD16, CD57, CD127, CD117, CD226 and NKp30) on the DN NK subset versus the CD11b^+^SP NK subset in TINK cells. Dot plots were gated on live NK cells using a lymphocyte gate based on forward scatter versus side scatter and an NK-cell gate identifying CD56^+^CD3^−^ cells. DN NK cells were identified based on gating for CD56^+^CD3^−^CD11b^−^CD27^−^, while CD11b^+^SP NK cells were identified based on a CD56^+^CD3^−^CD11b^+^CD27^−^ gate. Quadrants depicted were set on isotype controls. (B) The major phenotypic differences detected in the two subsets (DN and CD11b^+^SP NK) are summarised. Data shown represent the findings from 15 patients (mean±SD).

To assess the impact of the DN NK cells on TINK-cell maturation, we evaluated the phenotypes of both the DN and the CD11b^+^SP NK-cell subsets within the TINK-cell population. The proportions of the DN NK cells expressing CD16 and CD57 were significantly lower than those of the CD11b^+^SP NK cells. Moreover, CD127 (the IL-7 receptor α-chain) and CD117 (also known as KIT), two key markers of immature NK cells, were expressed at higher levels on DN NK cells than on CD11b^+^SP NK cells (Fig. 4A and B). These findings suggest that DN NK cells within the TINK-cell population display an immature phenotype.

To further dissect the roles of the DN and CD11b^+^SP NK-cell subsets of TINK cells, we assessed the expression of activating NK receptors on cells within the two subsets. We found that fewer DN NK cells were CD226^+^ or NKp30^+^ when compared with CD11b^+^SP NK cells (Fig. 4A and B). Hence, the DN NK-cell subset within human tumours exhibits an inactive phenotype, which is consistent with the immature developmental status of human DN NK cells reported in recent studies.

### DN NK cells have proliferative capacity and poor cytotoxic capacity

**Figure 5:**
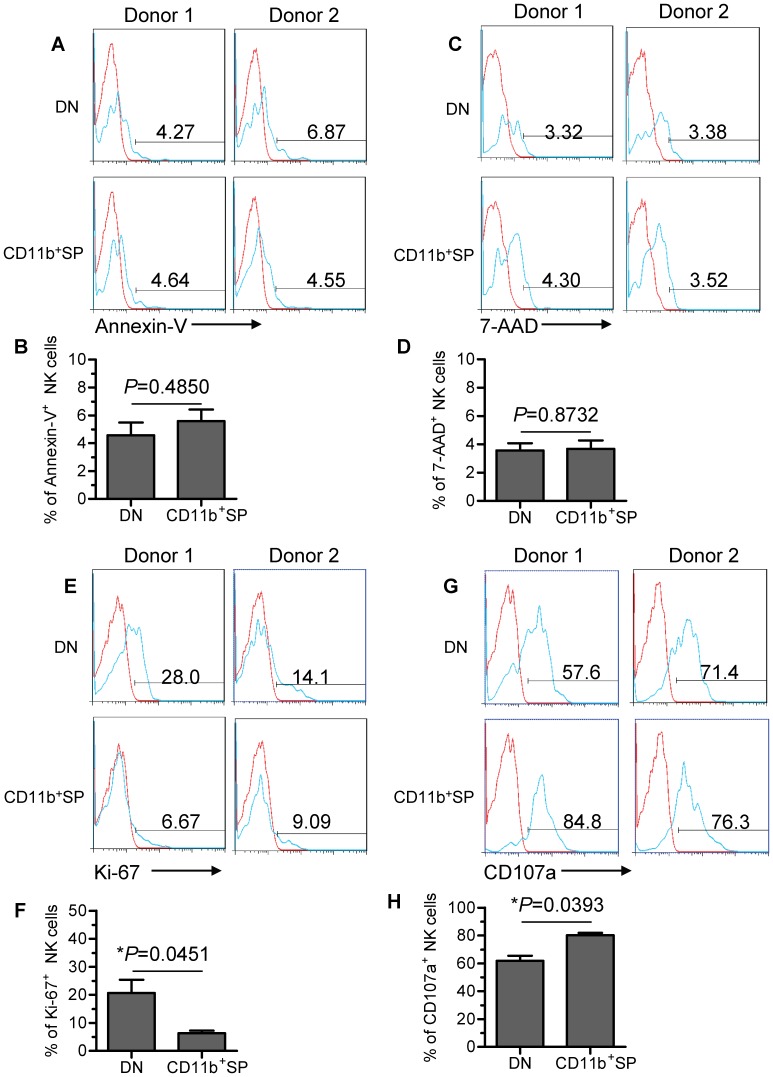
DN NK cells have proliferative capacity and poor cytotoxic capacity. (A and C) Two representative flow cytometry analyses of the expression of Annexin-V and 7-AAD (green graphs) relative to isotype-matched controls (red graphs) on the gated DN NK subset versus the CD11b^+^SP NK subset in TINK cells. (B and D) The frequency of Annexin-V^+^ and 7-AAD^+^ NK cells on two gated NK-cell subsets (n = 6; mean±SD). (E) Two representative flow cytometry analyses of Ki-67 expression (green graphs) relative to isotype-matched controls (red graphs) on the gated DN NK subset versus the CD11b^+^SP NK subset within TINK cells. (F) The frequency of Ki-67^+^ NK cells on two gated NK-cell subsets (n = 6; mean±SD). (G) Two representative flow cytometry analyses of CD107a expression (green graphs) relative to isotype-matched controls (red graphs) on the gated DN NK subset versus the CD11b^+^SP NK subset in TINK cells. (H) The frequency of CD107a^+^ NK cells on two gated NK-cell subsets (n = 6; mean±SD).

To investigate the potential developmental capability of DN and CD11b^+^SP subsets, we first evaluated the viability of the two subsets using Annexin-V and 7-Amino-Actinomycin D (7-AAD) staining. Minor proportions of the two subsets (<10%) were Annexin-V^+^ or 7-AAD^+^, indicating that both subsets were viable and not undergoing apoptosis (Fig. 5A–D). Furthermore, we found that the DN subset proliferated significantly more than the CD11b^+^SP subset as determined by higher Ki-67 expression (Fig. 5E and F), indicating that the DN NK-cell subset has developmental potential.

Furthermore, we evaluated the cytotoxic capacity of the two NK-cell subsets by analysing the expression of CD107a, a functional marker that identifies NK cell-mediated lysis of target cells. In addition to the observed reduction in CD16 expression (Fig. 4A and B), the DN subset had lower CD107a expression than the CD11b^+^SP subset, suggesting reduced cytotoxicity of the DN subset (Fig. 5G and H). This observation is consistent with published report showing that CD11b can mediate cytotoxic priming by beta-glucan [39]. Hence, the DN NK-cell subset has a poor cytotoxic capacity. We also measured the cytotoxic capacity of TINK cells compared with pNK cells from autologous patients by analysing the expression of CD107a. Diminished CD107a staining was observed on TINK cells (Fig. S1), suggesting reduced cytotoxicity for these cells, which is consistent with the previous study [13].

### The appearance of DN NK cells is associated with tumour progression in humans

**Figure 6:**
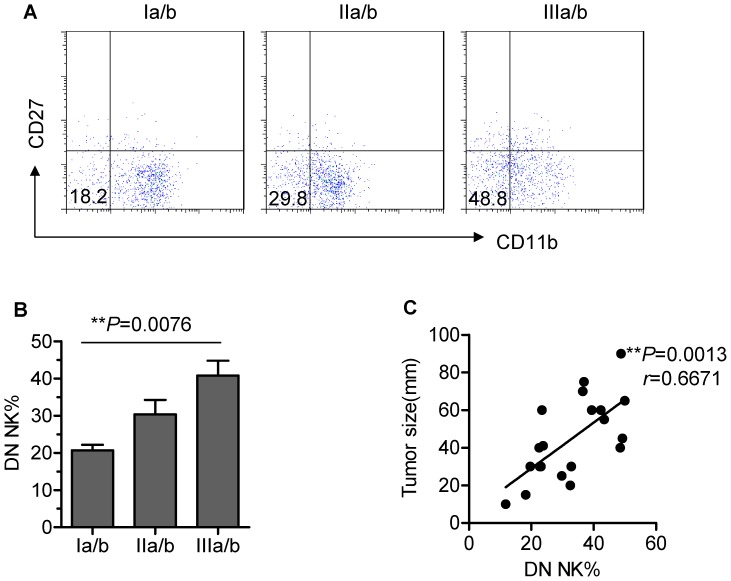
The frequency of tumour-infiltrating DN NK cells is highly associated with clinical outcome. (A) Representative flow cytometry analysis of the expression of CD27 and CD11b on TINK cells from NSCLC patients with tumours of distinct stage classifications based on tumour node metastasis (TNM). Dot plots were gated on live NK cells using a lymphocyte gate based on forward scatter and side scatter and an NK-cell gate (CD56^+^CD3^−^). DN NK cells were analysed by gating on CD56^+^CD3^−^CD11b^−^CD27^−^ cells. Quadrants depicted were set on isotype controls. (B) The frequency of tumour-infiltrating DN NK cells is correlated with the malignant progression of lung carcinoma (n = 20; mean±SEM). (C) The frequency of DN NK cells within tumours is directly correlated with tumour size. The *y*-axis represents the maximum diameter of the resected tumours.

To determine the functional role of DN NK cells in tumours, we evaluated the influence of DN NK cell presence on clinical outcomes. The NSCLC patients included in this study were classified into 3 groups (Ia/b, IIa/b and IIIa/b) based on tumour node metastasis (TNM). The clinical characteristics of the patients are summarised in Table S1. We first determined that a negative correlation exists between the absolute counts of total TINK cells and tumour size and progression stage (Fig. S2). Furthermore, a representative phenotypic analysis of CD11b and CD27 expression in TINK cells isolated from tumours of 3 different stages is depicted in Figure 6A. Remarkably, the frequency of DN (CD11b^−^CD27^−^) NK cells within the TINK-cell population increased as tumours progressed (Fig. 6B). We next examined the relationship between the frequency of DN NK cells and the size of the tumours. Notably, the frequency of DN NK cells within the TINK-cell population positively correlated with the maximum diameter of the resected tumours (Fig. 6C). These observations indicate that DN NK cells play an important role in tumour progression. We further investigated overall survival (OS) curve by Kaplan-Meier method. The patients included were classified into 3 groups (Low, Median and High DN NK%) based on the frequency of DN NK cells. Analysis of Kaplan-Meier survival curve showed a significant difference between the groups with low DN NK% and high DN NK% (Fig. S3), suggesting that the frequency of DN NK cells is associated with clinical outcome.

### The kinetics of DN NK-cell accumulation in lung tissue is associated with tumour progression in vivo

**Figure 7:**
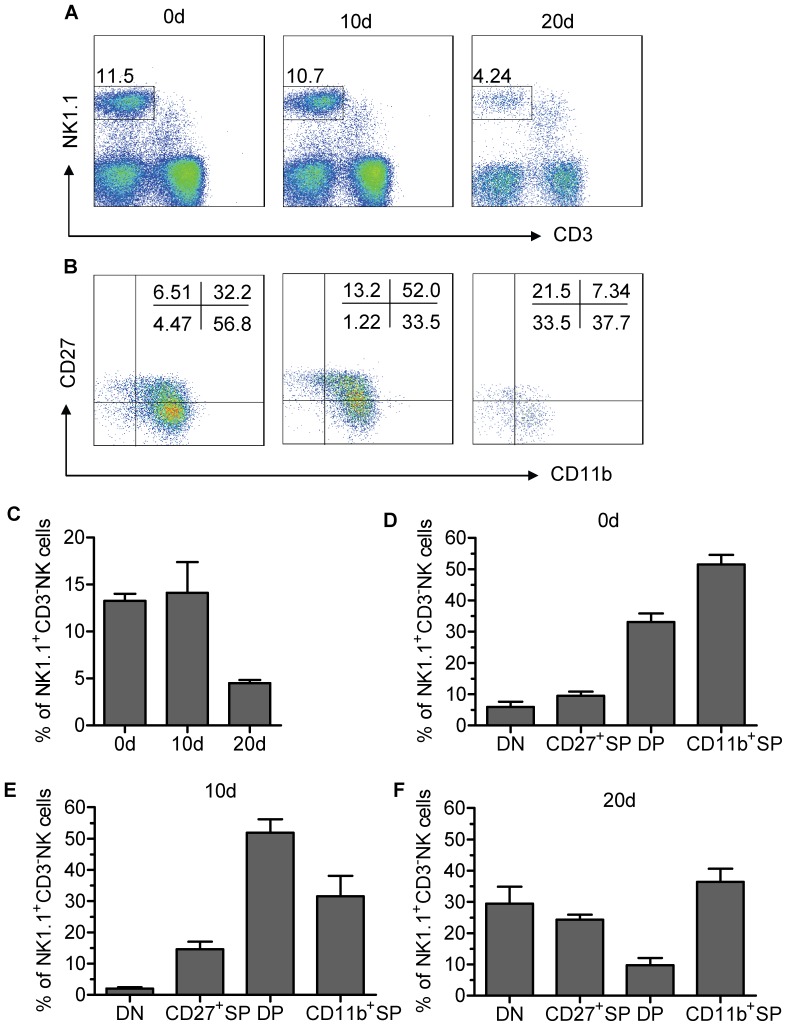
The kinetics of DN NK-cell accumulation in tumours is associated with tumour progression in vivo. (A) To establish tumours, C57BL/6 mice were intrapleurally injected with 5×10^5^ live Lewis lung cancer (LLC) cells. Representative flow cytometry analysis of lung TINK cells at different time-points after LLC injection. Dot plots were gated on live NK cells using a lymphocyte gate based on forward scatter versus side scatter and an NK-cell gate using NK1.1^+^CD3^−^ cells. Quadrants depicted were set on isotype controls. (B) Representative flow cytometry analysis of CD27/CD11b expression in lung TINK cells at different time-points after LLC injection. Quadrants depicted were set on isotype controls. (C) The frequency of TINK cells in lung tissue isolated from C57BL/6 mice at each time-point (n = 6 each). (D–F) The frequency of each subset of lung TINK cells isolated from C57BL/6 mice at each time-point (n = 6 each). All experiments were performed three times with similar results.

To further confirm the correlation between the frequency of tumour-infiltrating DN NK cells and the progression of lung carcinoma, we examined the expression of CD11b and CD27 on TINK cells from lung tissue in an intrapleurally implanted murine Lewis lung cancer (LLC) model [40]. Consistent with previous reports, the frequency of TINK cells in the lung tissue gradually decreased over time following LLC injection (Fig. 7A and C). Analysis of the frequency of each NK-cell subset within the TINK-cell population at different time-points revealed several findings. We observed striking differences in the frequency of each subset within the lung TINK-cell population at each time-point (here depicted as 0 d, 10 d and 20 d) (Fig. 7B). The injection of LLC induced a reduction in the proportion of CD11b^+^SP NK cells within the lung tissue and a concomitant increase in the proportion of DN NK cells over time (Fig. 7D–F). Collectively, our findings suggest that the frequency of DN NK cells within the TINK-cell population is associated with the progression of lung carcinoma.

To determine whether the tumour microenvironment was responsible for the accumulation of tumour-infiltrating DN NK cells, we measured the expression of ICAM-1, a ligand for CD11b/CD18 (also referred to as Mac-1 and CR3), on tumour cell lines. Human adenocarcinoma (ADC)-derived A549 cells and mouse LLC cells expressed minimal surface ICAM-1 (Fig. S4A). In contrast, high concentrations of soluble ICAM-1 (sICAM-1) were detected in malignant pleural fluid samples and A549 supernatants compared with plasma from NSCLC patients and healthy controls (Fig. S4B), implying that the level of sICAM-1 is high in the tumour microenvironment. Thus, we hypothesise that the secretion of sICAM-1 by human tumours may hinder the interaction between ICAM-1 and CD11b/CD18, leading to the downregulation of CD11b expression on NK cells and thereby resulting in the DN NK-cell accumulation.

## Discussion

Analogous to adaptive immunity, NK cell-mediated immunity can be divided into distinct developmental and effector phases. Although recent studies have described the developmental stages of NK cells [29], [30], [33], the relationship between NK-cell development and function, especially in the context of disease, remains poorly understood. In this study, we present several lines of evidence indicating that tumour-infiltrating NK cells have a unique distribution of NK-cell subsets, due to the effects exerted by the tumour microenvironment.

Our prior studies have shown that CD11b and CD27 expression defines NK-cell maturation status, reflecting a developmental continuity of human NK cells [33]. By investigating the expression of CD11b and CD27 on TINK cells from NSCLC patients, we characterised the maturation status of tumour-infiltrating NK-cell subpopulations. Interestingly, we found a substantial CD11b^−^CD27^−^ NK-cell subset within the TINK-cell population but not within the pNK-cell population from the same patients or from healthy control subjects. However, the mechanism by which such a large DN NK-cell subset accumulates within a tumour remains unsolved. One possibility is a higher rate of active death for the mature NK cells (CD11b^+^SP NK subset) than for the immature NK cells (DN NK subset) within the tumour, leading to a relative accumulation of tumour-infiltrating DN NK cells. However, we showed that DN and CD11b^+^SP subsets display equal viability, thus ruling out this possibility (Fig. 5A–D).

It is also possible that the tumour microenvironment could induce NK cells of an immature phenotype, just as a previous study indicated that the phenotypic alterations of TINK cell could be induced by the breast tumour microenvironment [13]. However, the mechanism by which the tumour microenvironment influences TINK-cell maturation remains unclear. Although little is known about NSCLC-infiltrating NK cells, in situ NK cells have been investigated in well-conducted studies [11]–[13]. NK cells are recruited to the tumour environment, where they are localised mainly to the tumour stroma rather than the tumour nest, implying that TINK cells are not in direct contact with cancer cells. The lack of physical contact between TINK cells and tumour cells suggests that tumour cells may produce soluble factors that influence TINK-cell development [41]. TGF-β is a likely candidate [13], [42] because it is known to down-regulate GATA-3, and GATA-3-deficient bone marrow NK cells display a CD11b^low^ phenotype [43]. Thus, we assessed TGF-β levels in malignant pleural fluid samples and supernatants from NSCLC-derived A549 cells by ELISA. However, the levels of TGF-β detected in these samples were not significantly different from those detected in plasma samples from NSCLC patients and healthy control subjects (data not shown). We thus searched for another soluble factor that could affect TINK-cell maturation. Previous studies have shown that the expression of ICAM-1, a ligand for CD11b/CD18 (also referred to as Mac-1 and CR3), is frequently reduced or absent in NSCLC with tumour cell dissemination [44], [45]. Accordingly, A549 and LLC cells expressed minimal surface ICAM-1. However, high concentrations of soluble ICAM-1 (sICAM-1) were detected in malignant pleural fluid samples and A549 supernatants, implying that the level of sICAM-1 is high in the tumour microenvironment. The interaction between ICAM-1 and CD11b/CD18 integrin plays a critical role in leukocyte trafficking, immune synapse formation and co-stimulation [46], [47]. Moreover, expression of CD11b is associated with NK-cell effector function [39]. Analogous to the effect of sMICA on NK-cell function [48], [49], it is possible that the secretion of sICAM-1 by human tumours may hinder the interaction between ICAM-1 and CD11b/CD18, leading to the downregulation of CD11b expression on NK cells and thereby affecting the TINK-cell functional maturation. However, elevated levels of sICAM-1 only partially explain the presence of immature NK cells within the tumour. Therefore, we cannot rule out the possibility that other soluble factors produced by tumour cells contribute to this process. Our findings demonstrate that the tumour microenvironment may render TINK cells less tumouricidal and thereby contribute to cancer progression, as CD11b downregulation was detected on TINK cells but not on pNK cells from the NSCLC patients. An alternate mechanism that could account for our findings would be that immature NK might be attracted to infiltrate tumour tissue, as opposed to an active role of tumour tissue in stimulating the loss or reversal of NK maturity. Future studies are required to elaborate on the mechanism by which the tumour microenvironment affects the functional maturation of TINK cells.

Numerous studies have examined the relationship between tumour-infiltrating NK cells and clinical outcomes. One study demonstrates that the frequency of NK cells within NSCLC tumours directly correlates with the size of the resected tumours [12]. Strikingly, our results highlight the important role of DN NK cells in tumour progression, not only because the frequency of DN (CD11b^−^CD27^−^) NK cells within the TINK-cell population is clearly correlated with tumour stage and tumour size, but also because DN NK cells have developmental potential.

In conclusion, our study has provided new insights into NK-cell maturation within human tumours. To the best of our knowledge, this is the first report of a significant immature CD11b^−^CD27^−^ NK-cell subset within the tumour-infiltrating NK-cell population and of a correlation between DN NK-cell frequency and tumour progression. More importantly, our data provide evidence that the tumour microenvironment may render TINK cells less tumouricidal and thereby contribute to cancer progression.

## Materials and Methods

### Ethics Statement

The study was approved by the Institutional Review Board of the University of Science and Technology of China. Human primary lung tumour tissue was obtained during surgery from 38 treatment-naive patients with NSCLC. Peripheral blood samples were collected from the same patients prior to surgery. In accordance with the Declaration of Helsinki and the Institutional Review Board of the University of Science and Technology of China, all participants provided written informed consent, which was obtained before enrolment in the study.

The study was carried out in strict accordance with the recommendations in the Guide for the Care and Use of Laboratory Animals. The animal experiment facilities were approved by the Anhui Provincial Department of Science and Technology, the approval ID is SYXK (Anhui) 2005-004. All protocols involving animal work were approved by the Ethics Committee of Animal Experiments of the University of Science and Technology of China (Permit Number: USTCACUC1201051). All surgery was performed under anaesthesia, and all efforts were made to minimize animal suffering.

### Samples from patients and healthy control subjects

Peripheral blood and tumour tissue were acquired from the Department of Chest Surgery, Anhui Provincial Hospital (Hefei, China). Detailed patient characteristics are shown in Table S1. Peripheral blood samples were obtained from healthy donors at the Blood Centre of Anhui Province (Hefei, China) and used as controls. All of the samples were collected after obtaining written informed consent from the donors and following approval by the Institutional Review Board of the University of Science and Technology of China.

### Mice and in vivo tumour challenge

A total of 54 female C57BL/6 mice were purchased from Shanghai Experimental Animal Centre, Chinese Science Academy (Shanghai, China), and were maintained in a specific pathogen-free (SPF) facility under barrier conditions at Laboratory Animal Centre of the University of Science and Technology of China. All mice were fed a regular mouse chow and housed in normal night-day conditions under standard temperature and humidity. All animal studies were carried out at the biosafety laboratory of the Laboratory Animal Centre, University of Science and Technology of China. The experiment complied with the Animal Management Rule of the Ministry of Public Health, People's Republic of China (document No. 55, 2001), and the experiment protocol was approved by the Ethics Committee of Animal Experiments of the University of Science and Technology of China (Permit Number: USTCACUC1201051). All mice were used at 8–10 weeks of age [50], and were randomly divided into 3 groups (n = 6 each). One group of mice with no treatment were marked as control group (here depicted as 0 d group). To establish tumours [40], the other two groups of mice were intrapleurally injected with 5×10^5^ live Lewis lung cancer (LLC) cells in a volume of 0.1 mL phosphate buffered saline (PBS). The mice were examined daily after LLC injection. To assess tumour progression in the lung, one group of tumour cell-inoculated mice were sacrificed on 10 day following LLC injection, and the remaining group of tumour cell-inoculated mice were sacrificed on 20 day following LLC injection. Mice were sacrificed using CO_2_. This study was performed three times.

### Isolation of lymphocytes from samples

Primary lung tumours were washed extensively with phosphate-buffered saline (PBS) to remove peripheral blood lymphocytes and were processed by mincing the tissue with operative scissors. Mechanically disrupted tumour tissue was digested with 0.1% collagenase type IV (Sigma-Aldrich, St. Louis, MO) in serum-free RPMI 1640 for 1 hour at 37°C under agitation. After enzymatic digestion, single-cell suspensions were filtered and washed, and mononuclear cells (MNCs) were purified using Ficoll-Hypaque density gradient centrifugation.

Similarly, peripheral blood mononuclear cells (PBMCs) were isolated from blood samples by Ficoll centrifugation.

To isolate mononuclear cells (MNCs), tumour-bearing mice were euthanised, and the lung tissues were collected. The tissues were mechanically disrupted and then digested with collagenase I (Sigma-Aldrich, St. Louis, MO) to create single-cell suspensions. After washing, the cell pellets were resuspended in 40% Percoll (GE Healthcare, Uppsala, Sweden), gently overlaid onto 70% Percoll, and centrifuged at room temperature. MNCs were isolated from the Percoll interface.

### Flow cytometric analysis of NK-cell phenotypes

Lymphocytes from human blood/tumour samples were incubated for 30 minutes at 4°C with the following mouse anti-human monoclonal antibodies: anti-CD3, anti-CD56, anti-CD27, anti-CD11b, anti-CD16, anti-CD57, anti-CD127, anti-CD117, anti-NKG2A, anti-NKp30 and anti-CD226 (all from BD Bioscience, San Jose, CA). Mouse serum was used to block non-specific Fc-receptor (FcR) binding, and isotype-matched IgGs were used as negative control antibodies. FACS staining was performed according to the manufacturer's instructions. The FACS gating strategy is shown in Figure S5.

Mouse lung lymphocytes were prepared and stained with mAbs. FcRs were blocked with normal rat serum. Abs specific for the following Ags were used to stain murine lung lymphocytes: NK1.1, CD3, CD11b and CD27 (all from BD Bioscience, San Jose, CA).

The samples were analysed on a FACSCalibur flow cytometer (BD Biosciences). Flow cytometry data were analysed using FlowJo software (Tree Star, Inc., Ashland, OR).

### Apoptosis detection by Annexin-V and 7-AAD staining

Lymphocytes from human tumour samples were incubated with the following mouse anti-human monoclonal antibodies: anti-CD3, anti-CD56, anti-CD27 and anti-CD11b, as described above. After two washes in cold binding buffer (10 mM HEPES/NaOH [pH 7.4], 140 mM NaCl, 2.5 mM CaCl_2_), cells were stained with Annexin-V and 7-AAD (BD PharMingen) for 15 min at room temperature in the dark and analysed by flow cytometry.

### Ki-67 and CD107a detection

Lymphocytes from human tumour samples were incubated with the following mouse anti-human monoclonal antibodies: anti-CD3, anti-CD56, anti-CD27, anti-CD11b and anti-CD107a for 30 minutes at 4°C. After fixation and permeabilisation, cells were further stained with anti-CD107a Ab and anti-Ki-67 Ab for 1 h at 4°C, washed twice with permeabilisation buffer, and analysed by flow cytometry. Appropriate isotype Abs were used as controls for intracellular staining.

### Cell lines

Human adenocarcinoma (ADC)-derived A549 cells, obtained from the Chinese Academy of Sciences Cell Bank (Shanghai, China), were cultured in RPMI-1640 supplemented with 10% foetal calf serum (FCS) at a density of 10^7^ cells/ml. After incubation for 72 h, the culture supernatants were collected and assayed for cytokine levels by ELISA.

LLC cells derived from C57BL/6 mice with Lewis lung carcinoma, obtained from the Chinese Academy of Sciences Cell Bank (Shanghai, China), were cultured in DMEM supplemented with 10% FCS. The cells were cultured for less than 6 months after resuscitation. Early-passage cells were used for all experiments and were not reauthenticated.

### Cytokine detection by ELISA

To measure cytokine levels in NSCLC patients, plasma samples were collected from all patients and healthy control subjects and stored at −70°C until use. Likewise, malignant pleural fluid samples and A549 supernatants were also appropriately collected and stored before utilisation. TGF-β and sICAM-1 were assayed using standard sandwich ELISA kits (CUSABIO BIOTECH CO., LTD.) according to the manufacturer's instructions.

### Statistical analyses

We used two-tailed paired Student's t-tests (difference between two groups) or two-tailed unpaired Student's t-tests to determine the statistical significance (*P≤0.05; **P≤0.01; ***P≤0.001). Overall survival (OS) curve was estimated by Kaplan-Meier method and differences between the groups of patients were evaluated using the log-rank test at minimal *P* value.

## Supporting Information

Figure S1 **TINK cells had poor cytotoxic potential compared with paired pNK cells.** (A) Two representative flow cytometry analyses for CD107a expression (green graphs) relative to isotype-matched controls (red graphs) on TINK cells as compared with that on pNK cells from autologous patients. (B) The frequency of CD107a^+^ NK cells within the above-mentioned two NK-cell populations (n = 6; mean±SD).
(TIF)

Figure S2 **The absolute counts of TINK cells are highly associated with tumour progression.** (A) Absolute counts of TINK cells are negatively correlated with the malignant progression of lung carcinoma (n = 20; mean±SEM). (B) Absolute counts of TINK cells are negatively correlated with tumour size. The *y*-axis represents the maximum diameter of the resected tumours.
(TIF)

Figure S3 **Kaplan-Meier curve of overall survival (OS).** The patients included were classified into 3 groups (Low, Median and High DN NK%) based on the frequency of DN NK cells. The group with low DN NK% (black curves), median DN NK% (green curves) and high DN NK% (red curves) are shown.
(TIF)

Figure S4 **sICAM-1 levels are elevated in malignant pleural fluid samples and A549 supernatants.** (A) Representative flow cytometry analysis of ICAM-1 expression (green graphs) relative to isotype-matched controls (red graphs) on the surface of A549 and LLC cells. (B) Levels of sICAM-1 in plasma samples from healthy donors and NSCLC patients, malignant pleural fluid samples and A549 supernatants were quantified by sandwich ELISA.
(TIF)

Figure S5 **FACS gating strategy.** (A) The FACS gating strategy for excluding dead and irrelevant cells. (B) The FACS gating strategy for isolating total CD3^-^CD56^+^ TINK cells within the lymphocyte gate.
(TIF)

Table S1 Clinical characteristics of 38 patients with NSCLC. NOTE: Pathologic staging and histologic types of lung cancer were determined according to the TNM staging system and the histologic classification of the World Health Organization, respectively.
(TIF)
