# Supplementary figures and images for: CD11b−CD27− NK Cells Are Associated with the Progression of Lung Carcinoma
Source: PLoS One. 2013 Apr 2;8(4):e61024. doi: 10.1371/journal.pone.0061024 (PMC3614924; doi:10.1371/journal.pone.0061024)

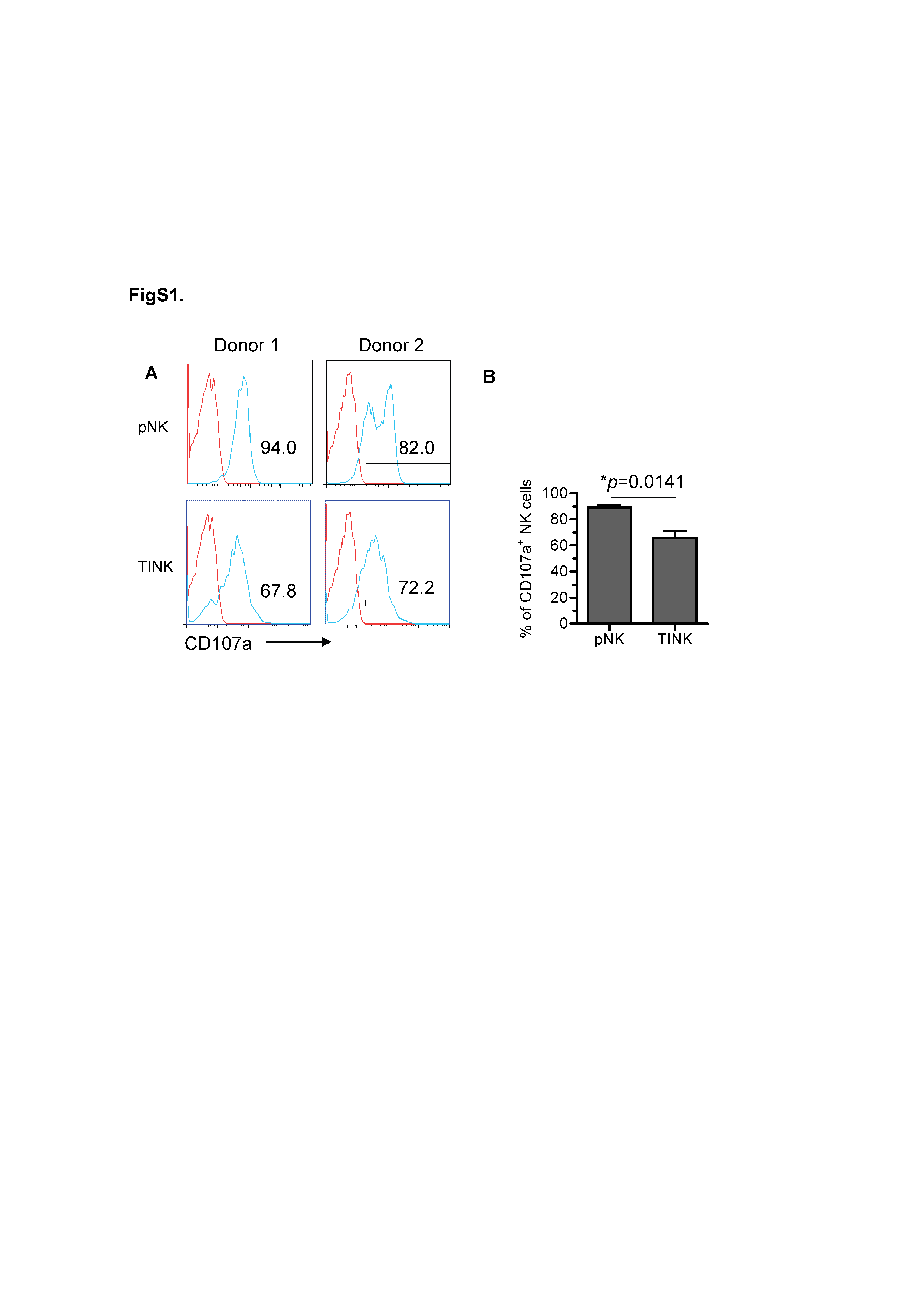

Supplement: Figure S1 — TINK cells had poor cytotoxic potential compared with paired pNK cells. (A) Two representative flow cytometry analyses for CD107a expression (green graphs) relative to isotype-matched controls (red graphs) on TINK cells as compared with that on pNK cells from autologous patients. (B) The frequency of CD107a+ NK cells within the above-mentioned two NK-cell populations (n = 6; mean±SD). (TIF) [file pone.0061024.s001.tif]

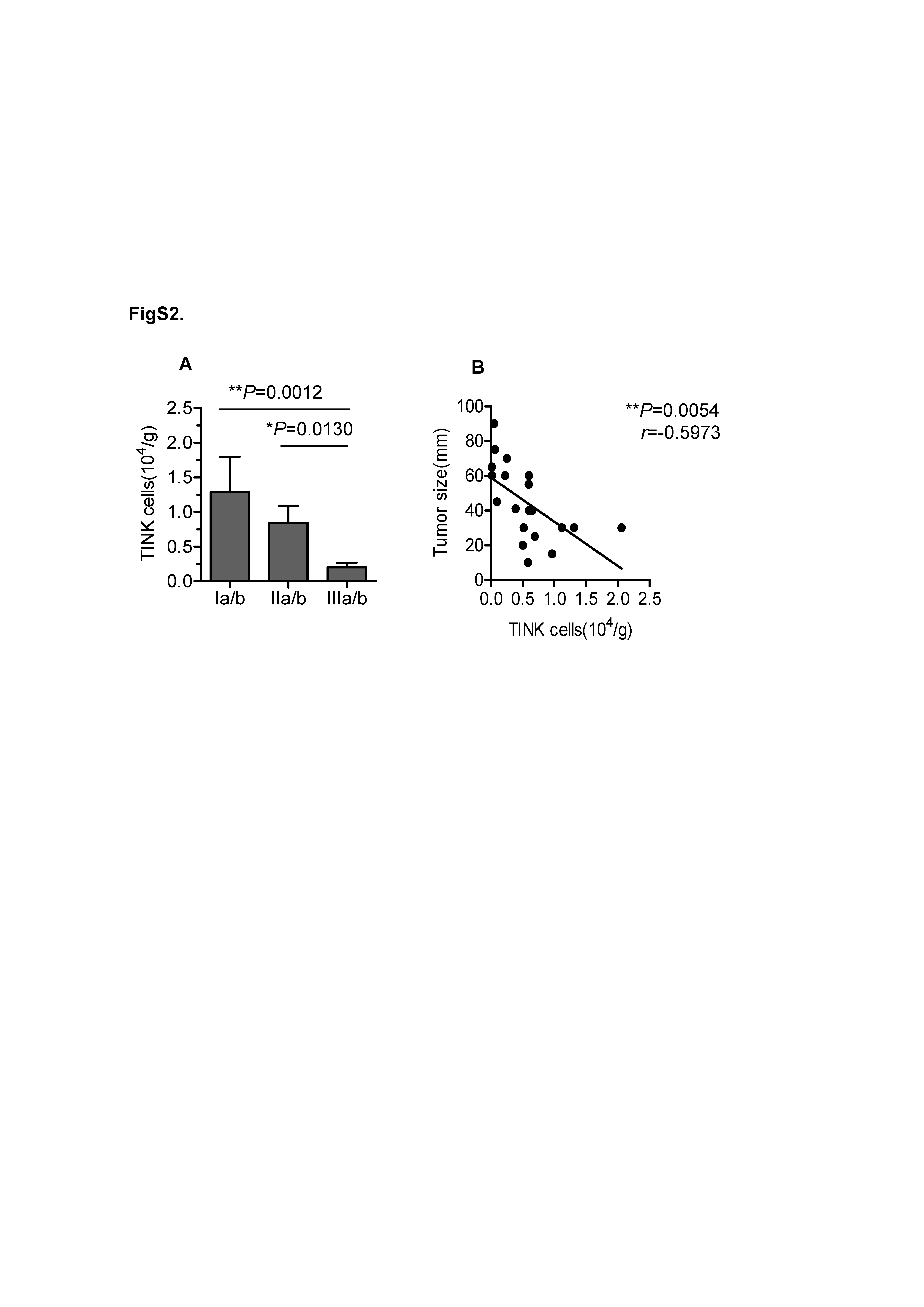

Supplement: Figure S2 — The absolute counts of TINK cells are highly associated with tumour progression. (A) Absolute counts of TINK cells are negatively correlated with the malignant progression of lung carcinoma (n = 20; mean±SEM). (B) Absolute counts of TINK cells are negatively correlated with tumour size. The y-axis represents the maximum diameter of the resected tumours. (TIF) [file pone.0061024.s002.tif]

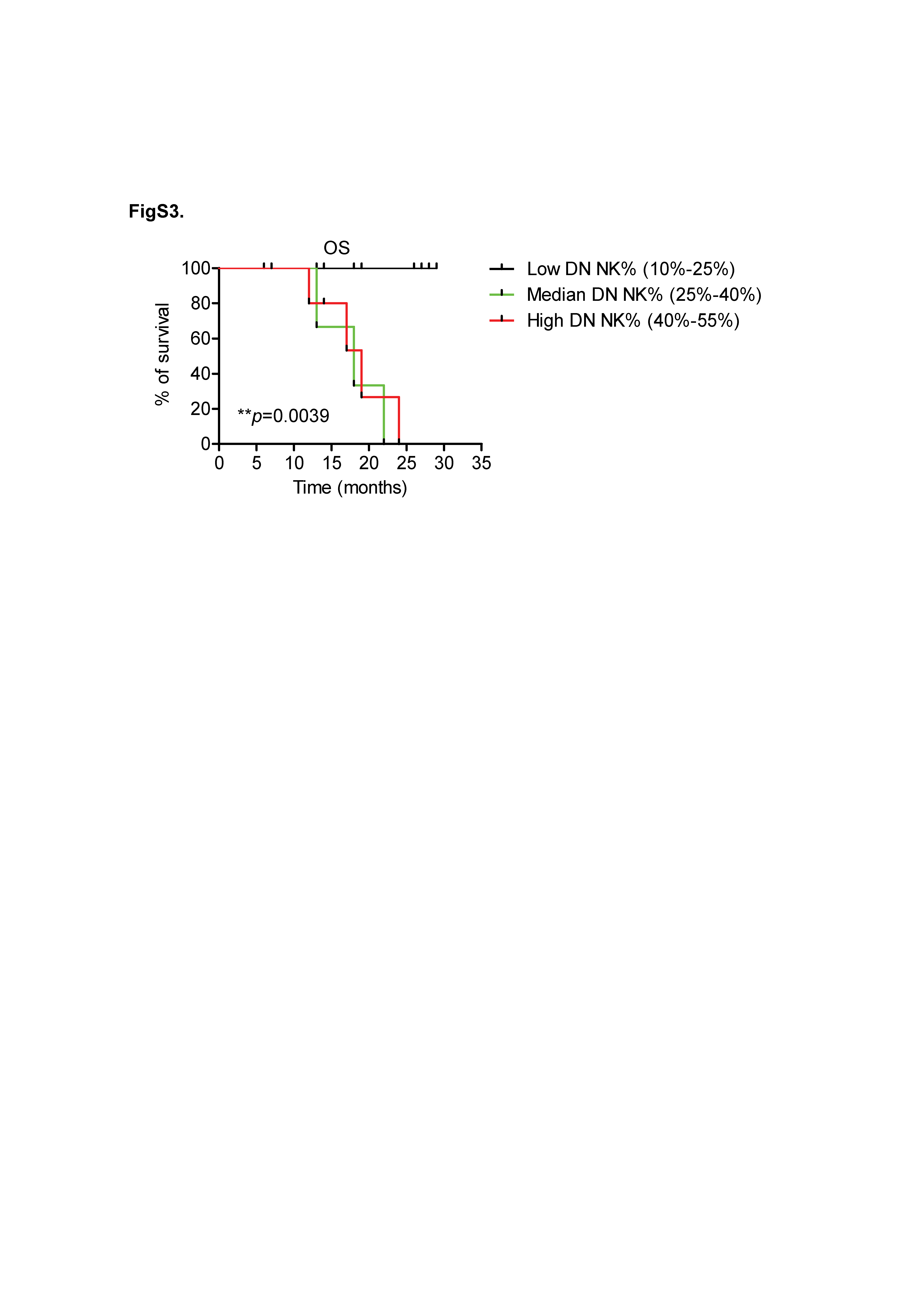

Supplement: Figure S3 — Kaplan-Meier curve of overall survival (OS). The patients included were classified into 3 groups (Low, Median and High DN NK%) based on the frequency of DN NK cells. The group with low DN NK% (black curves), median DN NK% (green curves) and high DN NK% (red curves) are shown. (TIF) [file pone.0061024.s003.tif]

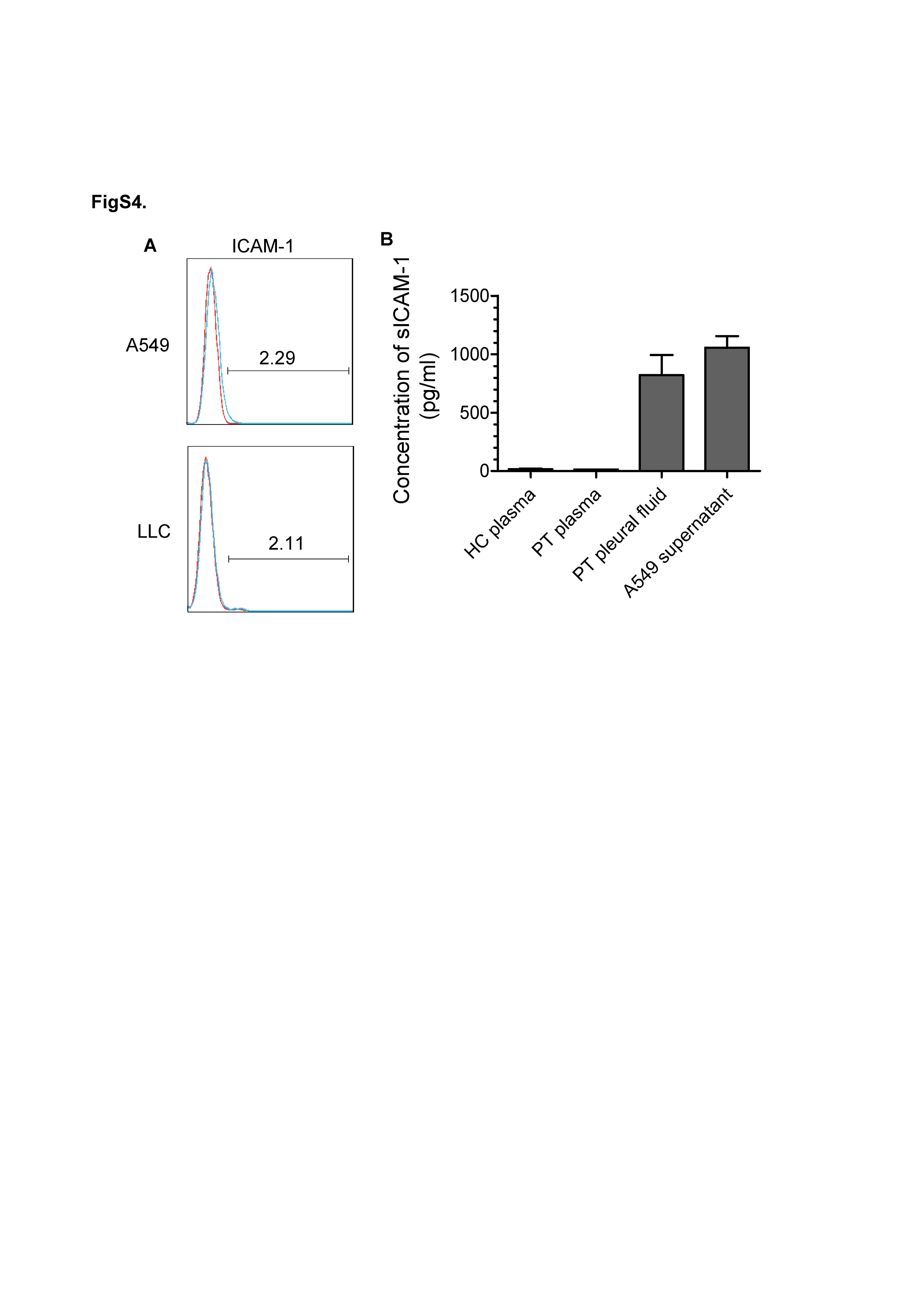

Supplement: Figure S4 — sICAM-1 levels are elevated in malignant pleural fluid samples and A549 supernatants. (A) Representative flow cytometry analysis of ICAM-1 expression (green graphs) relative to isotype-matched controls (red graphs) on the surface of A549 and LLC cells. (B) Levels of sICAM-1 in plasma samples from healthy donors and NSCLC patients, malignant pleural fluid samples and A549 supernatants were quantified by sandwich ELISA. (TIF) [file pone.0061024.s004.tif]

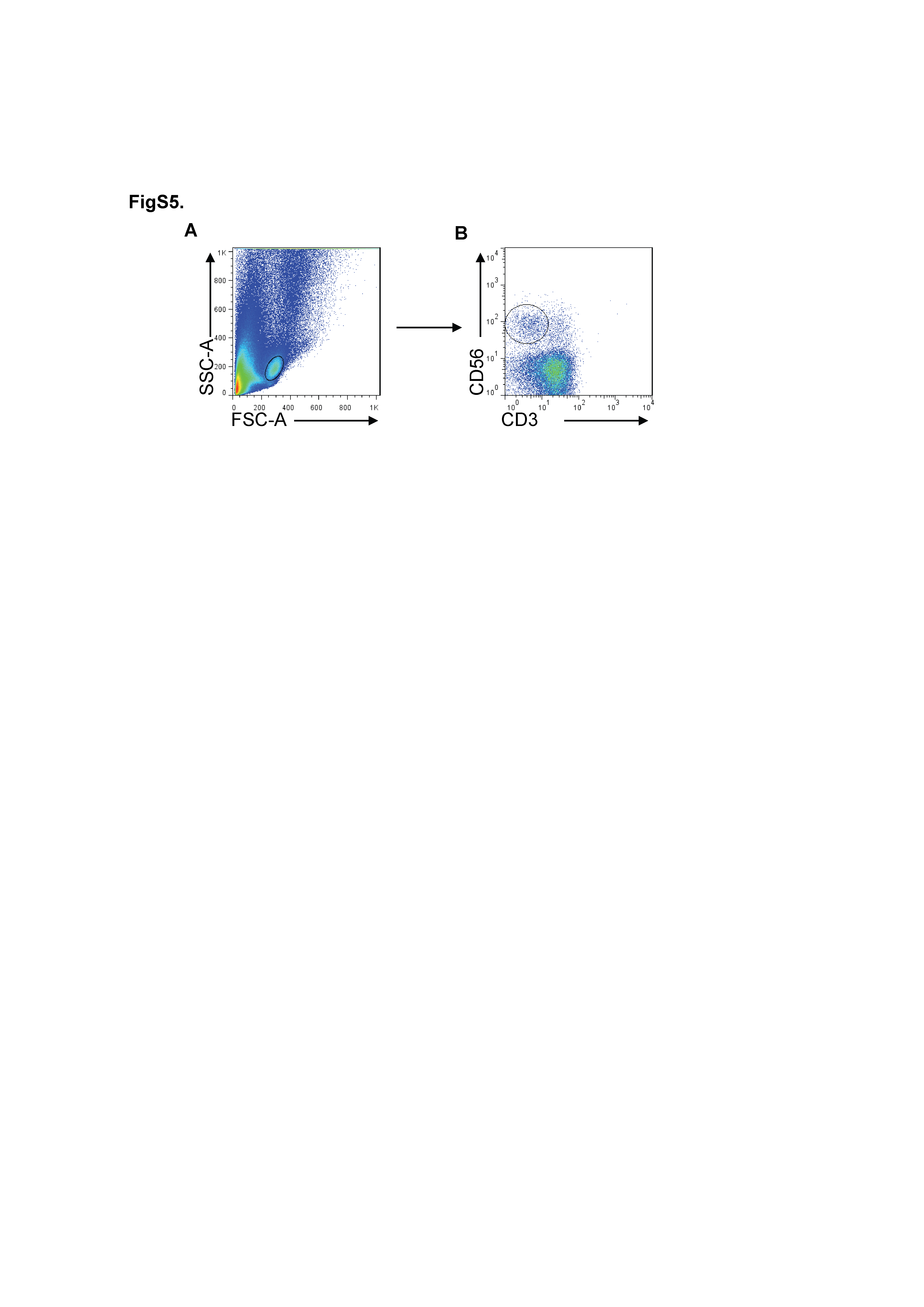

Supplement: Figure S5 — FACS gating strategy. (A) The FACS gating strategy for excluding dead and irrelevant cells. (B) The FACS gating strategy for isolating total CD3-CD56+ TINK cells within the lymphocyte gate. (TIF) [file pone.0061024.s005.tif]
